# How sleep redraws phonemic categories after auditory selective adaptation

**DOI:** 10.3758/s13423-025-02819-x

**Published:** 2026-01-14

**Authors:** Nicolas Dumay, Arthur G. Samuel

**Affiliations:** 1 Department of Psychology, University of Exeter, Perry Road, Exeter, EX4 4QG UK; 2 Basque Center on Cognition, Brain and Language, Donostia/San Sebastián, Spain; 3 IKERBASQUE, Basque Foundation for Science, Bilbao, Spain; 4 Department of Psychology, Stony Brook University, Stony Brook, NY USA

**Keywords:** Selective adaptation, Sleep, Memory consolidation, Speech perception, Phonemic categories, Perceptual learning, Frequency effects

## Abstract

After information has either been perceived or brought into working memory from long-term memory, it may remain active for hours or days. There is extensive evidence that sleep can consolidate newly learned material into long-term memory, and some recent work shows that sleep may also help clear out either unneeded or already established information. We examine the effect of sleep on a third type of information: adjustments to established speech categories caused by repeated exposure to a speech sound—selective adaptation. We find that sleep does *not* consolidate selective adaptation per se. Instead, sleep implements a change in phoneme category frequency to reflect the properties of the input—the many instances of the adapting sound that had been presented repeatedly. While adaptation temporarily reduces the perception of tokens similar to the repeating sound, sleep increases their perception, producing a “reverse adaptation” pattern. The results constrain models of phoneme category adjustment, favoring those that have separate mechanisms for assimilative versus contrastive effects over those with a single mechanism for both types of effects.

**Supplementary Information:**

The online version contains supplementary material available at 10.3758/s13423-025-02819-x.

## Introduction

As we go about our daily activities, much of what we do can be characterized as some mixture of perception, memory, and action: We take in the world around us through our senses, we try to make sense of these perceptions given what we know, and we act upon them if necessary. To do this, we continuously invoke a set of long-term procedural codes to organize perceptual inputs and meet the goals of our actions. From this perspective, perception and action are thus inherently intertwined with memory.

In the moment, perceptual inputs and relevant long-term knowledge are pulled together in working memory and exploited. Most of the contents created in working memory will never make it into long-term memory, as most of what we perceive and do will be useful at the time, but not in the long term. Indeed, the assumption has always been that most of these short-term representations should be “silenced” as soon as the current perceptual, cognitive, or expressive challenge has been resolved. However, many of these codes appear to still be active well after the event, and continue to influence how we behave for quite some time before fading away. Here, we focus on the by-products of perception, and ask whether sleep alters their fate in any way.

A perceptual phenomenon that lingers for hours and may reflect adjustments to long-term procedural codes is “auditory selective adaptation” (Eimas & Corbit, 1973). In selective adaptation, repeated exposure to a sound (i.e., the “**adaptor**”) results in listeners perceiving that sound less often on a posttest compared to a pretest. For instance, after many exposures to an unambiguous /wa/, listeners hear fewer instances on a test continuum between /ba/ and /wa/, as “wa” (i.e., they report hearing “ba” more often than at pretest). This shift in the identification function is usually seen as a type of perceptual learning for speech: If the speech environment is heavily biased in some way, the perceptual system requires stronger acoustic cues to perceive the dominant sound. In a recent study (Samuel & Dumay, 2021), we tracked the dissipation of selective adaptation in awake participants. We found that identification shifts were mostly intact after 25 min, much smaller but still present after 90 min, and still measurable after 5 h and 30 min.

Since Karni et al.’s (1994) seminal sleep-deprivation experiments showing that REM-sleep benefits visual texture discrimination, there have been many demonstrations that sleep stabilizes, or even sharpens, recently learned and likely slowly degrading, perceptual skills (e.g., Atienza et al., 2004; Brawn et al., 2010; de la Chapelle et al., 2022; Earle & Myers, 2015; Earle et al., 2017; Fenn et al., 2003; Mednick et al., 2002; Stickgold et al., 2000; Tamaki et al., 2020; for a review, see Nusbaum et al., 2018). Given that selective adaptation is still detectable after almost 6 h in the awake state, the phenomenon provides an ideal testbed for examining how sleep might affect the leftovers of perceptual activity.

The literature on perceptual learning and speech has barely touched on the role of postlearning sleep. Fenn et al. (2003) provide the closest case to what we are testing. The authors trained listeners to comprehend poor-quality synthetic speech, by giving participants a transcript of what was being said. They found that the effect of sleep depended on its timing: When it came soon after training, sleep made the new skill resistant to subsequent daytime interference; in contrast, when it was delayed by 12 h in the awake state, it restored the benefits of training that had degraded in the interval. Compared with auditory selective adaptation, however, the use of whole-word synthetic speech makes it is impossible to know exactly what is being learned by the participants.

**Fig. 1:**
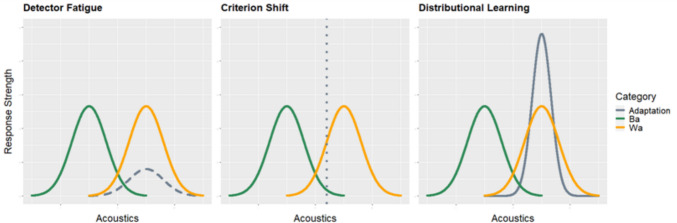
Schematic illustration of the three main accounts of auditory selective adaptation. Left: Eimas and Corbit’s (1973) feature detector fatigue/habituation. Middle: Diehl et al.’s (1980) contrast-induced criterion shift. Right: Kleinschmidt and Jaeger’s (2015) distributional learning. The orange and green curves represent the strength of response for the adapting category (e.g., /wa/) and the competing category (e.g., /ba/) before adaptation. The grey curves/lines represent the presleep adjustments resulting from adaptation. In the right panel, the continuity of the grey line indicates an actual alteration of long-term distributional (phonemic) knowledge. In other words, the strength of response faithfully reflects the underlying organization of sound categories in memory.

According to Kleinschmidt and Jaeger’s (2015, 2016) highly influential Bayesian account of speech perception, selective adaptation involves a change in the long-term distributional knowledge underlying phoneme categories. Their “belief-updating” model assumes that exposure continuously alters the distributional parameters of phonetic categories: thus, whereas exposure to an ambiguous token in the presence of disambiguating information enlarges the breadth (i.e., the variability) of the target category to include the token in question (as is the case in the phenomenon known as perceptual “recalibration”; Bertelson et al., 2003; Norris et al., 2003; Vroomen et al., 2007), repeated exposure to a prototype (as is the case in selective adaptation protocols) acts in the opposite fashion by sharpening the category around its mean (i.e., the adaptor). As is illustrated in the right panel of Fig. 1, this sharpening is precisely what shifts the crossover point toward the adaptor, thereby leading to the observed reduction in report of the adapted category.

The Kleinschmidt and Jaeger (2015, 2016) model explains adaptation through alterations of long-term statistical knowledge. As such, it fundamentally departs from earlier accounts of speech adaptation. The left panel of Fig. 1 depicts the sensory fatigue model as first proposed by Eimas and Corbit (1973). Before adaptation, detectors for the two relevant categories are in a kind of equilibrium. When one of the two detectors undergoes adaptation, however, its sensitivity gets reduced (i.e., *fatigued*) for a period of time, with the result that the crossover point is temporarily closer to the adaptor (as illustrated by the lower dashed curve). Similarly, the contrast-based, alternative model proposed by Diehl et al., (1978, 1980), and depicted in the middle panel of Fig. 1, does not assume alterations of core distributional parameters either. Adaptation is instead taken to reflect a contrast effect between the adaptor and the test items: the adaptor being typically drawn from either end of the test continuum, it represents a good, unambiguous exemplar of its phonetic category. Against this prototype, any test item situated near the category boundary will never sound as good, and must therefore belong to the other category, resulting in a criterion shift toward more conservative responses.

Because not all three accounts make the same, tacit predictions regarding a possible influence of postadaptation sleep, our results should clarify which account might be the most accurate. The contrast model, for instance, implies that participants have a good episodic memory of the phonetic properties of the adaptor at the time of test. As sleep should slow down forgetting the adaptor, this model predicts that adaptation should dissipate more slowly during sleep than during the same amount of time in the awake state. The distributional learning account should also be sensitive to consolidation during sleep. However, due to the procedural nature of the learning involved, here, sleep should make this learning more secure, or even promote it further despite the adaptor no longer being present. Thus, based on this account, a postsleep test should show perseveration of initial adaptation effects, if not their nontrivial amplification. In contrast, given the posited underlying mechanism, the sensory fatigue model, if anything, predicts the exact opposite: neural fatigue might actually clear more easily during sleep.

**Fig. 2:**
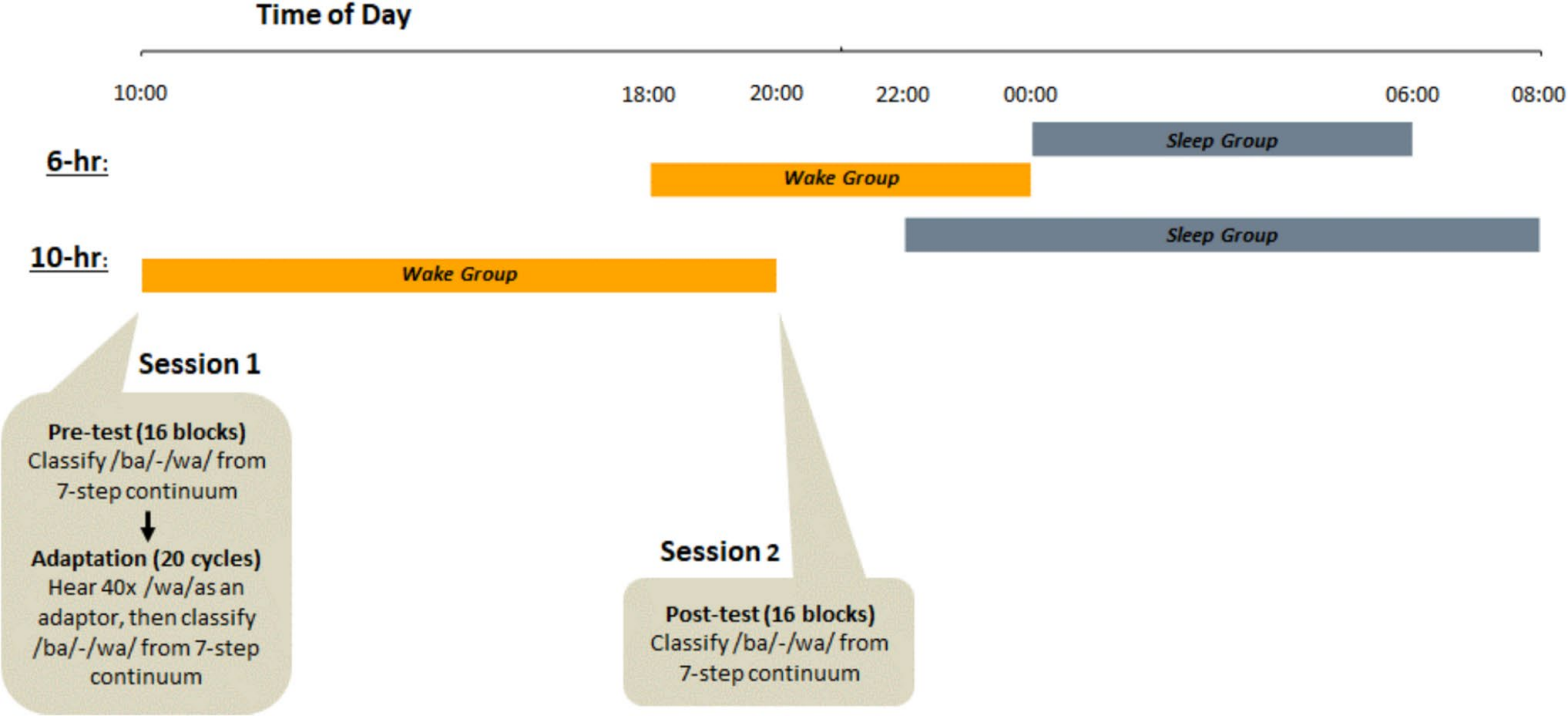
Sleep and Wake group intervals in the 6-h and the 10-h variations of the protocol. In the 6-h intervals, the Sleep group had Session 1 at midnight and Session 2 at 06:00; the Wake group had Session 1 at 18:00 and Session 2 at midnight. In the 10-h intervals, the Sleep group had Session 1 at 22:00 and Session 2 at 08:00; the Wake group had Session 1 at 10:00 and Session 2 at 20:00. As shown for the 10-h Wake group, in Session 1, participants first performed an identification pretest consisting of 16 randomizations of the seven steps of the /ba/ – /wa/ continuum; they then underwent 20 cycles of adaptation and identification. Each cycle consisted of 40 exposures to the /wa/ adaptor, followed by one randomization of the seven steps of the /ba/ – /wa/ continuum for identification. In Session 2, participants only performed the identification posttest, using the same procedure as the pretest.

To assess whether sleep alters the fate of adaptation shifts, we used the same stimuli as in Samuel and Dumay (2021)—we know that with these stimuli, adaptation should be almost gone after 6 h without sleep. Our study included two variations of a sleep/wake protocol (see Fig. 2), that used the same stimuli, and the same exposure and test procedures. In the **6-h variation**, a Sleep group underwent adaptation at midnight and was posttested at 06:00 the next morning, after a (short) night’s sleep. This was compared to a Wake group undergoing adaptation at 18:00 and posttested at midnight. Thus, the adaptation-to-posttest interval, as in Samuel and Dumay (2021), was about 5 h and 30 min. The **10-h variation** used a more “classic” PM-AM versus AM-PM protocol (e.g., Bäuml et al., 2014; Dumay, 2016, 2018; Dumay & Gaskell, 2007; Ekstrand, 1967; Monaghan et al., 2015; Payne et al., 2008). Here, adaptation and posttest occurred at 22:00 and at 08:00 the next morning for the Sleep group, versus 10:00 and 20:00 the same day for the Wake group.

The 6-h variation was motivated by our earlier findings and the need to have an interval long enough to allow participants to have decent sleep. In contrast, the 10-h variation allowed us to track the dissipation of adaptation in the awake state beyond 6 h, and provided the potential for an internal replication of sleep effects observed with the 6-h interval. Identification tests *during* adaptation served as time-of-day controls (see Mickes et al., 2023). As both groups in the 6-h variation were adapted in the evening, comparing the two variations of the protocol gave us a further check on any possible circadian influence.

## Methods

### Participants

A total of 116 native speakers of British English were tested (25 men; age range: 18–40, mean: 20 years 6 months). All were studying at the University of Exeter and none reported having any auditory, language, or sleep impairment, or being under medication altering sleep. For each variation of the protocol, participants were randomly assigned to either the Wake group or the Sleep group. Forty-three participants did the 10-h variation, with 22 participants assigned to the Wake group and 21 assigned to the Sleep group. The remaining 73 did the 6-h variation, with 38 participants assigned to the Wake group and 35 assigned to the Sleep group. We used a larger sample size in the 6-h than the 10-h variation because we assumed that sleep would have a weaker effect over the shorter adaptation-to-posttest interval. For practical reasons, the two variations were run consecutively rather than in parallel. Participants received either course credits or cash for taking part. None of the participants dropped out between the first and the second session. As detailed below, however, nine of them had their data excluded from the main analyses due to either poor sleep or poor discrimination of the endpoints of the test continuum.

### Statistical power

Sample size was based on Fenn et al.’s (2003) perceptual learning study, which found a significant sleep advantage of 8.7%. Based on this difference and a resulting effect size of .87, our power analysis (i.e., α = .05; β = .95; correlation between repeated measures = 0.5) in G*Power (Faul et al., 2009) indicated that a sample size of 20 would be sufficient to detect a similar effect using a repeated-measures analysis of variance (ANOVA).^1^ As we wanted to also detect a possible interaction with group (Sleep vs. Wake), for which Fenn et al. provided too few statistical details, we followed Fleiss’s (1986, Sect. 4.2) advice to test at least four (i.e., the number of cells implicated) times more participants than needed for a simple effect.

### Stimuli

The stimuli were a set of seven syllables that formed a continuum ranging from /ba/ to /wa/. They were taken from Samuel’s (1988) study and were also used in Samuel and Dumay’s (2021) investigation of how long adaptation shifts last (for participants remaining awake). As in the latter study, they were chosen because they produce strong and reliable immediate adaptation shifts. All stimuli were synthesized to be 300-ms long, with the slope and duration of formant transitions manipulated to go from the stop consonant /b/ at one end, to the continuant /w/ at the other end. Each step of the continuum differed from an adjacent step by 5 ms of transition duration, going from 25 ms of transition duration for the endpoint /b/ to 55 ms for the endpoint /w/. See Samuel (1988) for information about how these syllables were synthesized.

### Procedure

Participants came to the lab twice, either 10 h or 6 h apart. For the 10-h variation, the Wake group took an identification test (pretest) followed by the adaptation task, at 10:00, and returned to the lab to take the identification test again (posttest), at 20:00. The Sleep group followed the same procedure, but their first session was at 22:00 and their second session was at 08:00 the next morning. For the 6-h variation, the Wake group did their first session at 18:00 and their second at midnight; the Sleep group did their first session at midnight, and their second at 06:00 (see Fig. 2).

Participants were tested in sound-attenuated cubicles, with the stimuli presented binaurally at a comfortable listening level over Beyerdynamic DT-770 (80 Ω) Pro headphones. The *identification test* consisted of 16 randomizations of the 7-step /ba/ – /wa/ continuum (i.e., 16 × 7 = 112 trials). On each trial, participants had up to 5 s to categorize the presented syllable as either /ba/ or /wa/, by means of a key press. The next syllable followed 1 s later. This test took 6–7 min. The adaptation task differed from the identification test only by the presence of an adaptor sequence before each randomization of the test continuum. An adaptor sequence involved 40 presentations of the endpoint /wa/ syllable (i.e., the “adaptor”), with a 300-ms ISI after each presentation. The test included 20 blocks of adaptation and identification, with a 1-s interval between the final syllable of the adaptor sequence and the first syllable of the identification test. Participants were instructed to just listen during the adaptor sequence, and to only respond, using the usual procedure, when the test syllables were presented. This task took about 20 min.

Between the two sessions, the Sleep group wore a Philips Respironics Spectrum Plus actigraph and slept in their own bed. The actigraph tracked the participants’ movements in order to determine when they were (most likely) asleep and when they were not. The Wake group just went about their day as usual, but they were asked to refrain from napping if possible. At the second session, they all confirmed they had not.

### Data quality checks

First, based on the actigraphs’ data analyzed using the default parameters in the Philips® Actiware software, two participants—one in the 6-h and one in the 10-h variation—were excluded for sleeping much less than the rest of their group: both were more than 2.5 standard deviations below their group mean in terms of total sleep duration. Second, the results of the identification tests were examined to ensure our analyses only included participants who heard the test continuum as intended: In studies of this sort, a (small) proportion of participants produce identification functions that indicate that the person failed to hear differences between the test syllables. This is most evident by their failure to identify the endpoint /ba/ as “ba” and/or the endpoint /wa/ as “wa”. We computed the percent “wa” report averaged separately for Steps 1 and 2, and Steps 6 and 7, and set the minimum difference between these values to 41%—2 standard deviations below the mean (see Samuel, 2016, 2020; Samuel & Dumay, 2021, for a comparable procedure). Based on this procedure, seven participants were excluded from the final sample, leaving us with 39 participants in the 10-h variation (21 in the Wake group; 18 in the Sleep group) and 68 participants in the 6-h variation (37 in the Wake group; 31 in the Sleep group). Despite these slight differences in sample size *within* each variation on the protocol, the average time interval between the first session and the second session *across* variations was essentially the same for the Sleep and Wake groups (7 h 28 min [*SD* = 1 h 57 min] vs. 7 h 27 min [SD: 1 h 56 min]). Unsurprisingly, participants in the 10-h variation slept for longer (7 h 6 min, *SD* = 38 min) than those in the 6-h variation (4 h 45 min, *SD* = 28 min), a difference which a two-tailed *t* test showed was highly significant, *t*(47) = 14.66, *p* < .0001, Cohen’s *d* = 4.34.

## Results

**Fig. 3:**
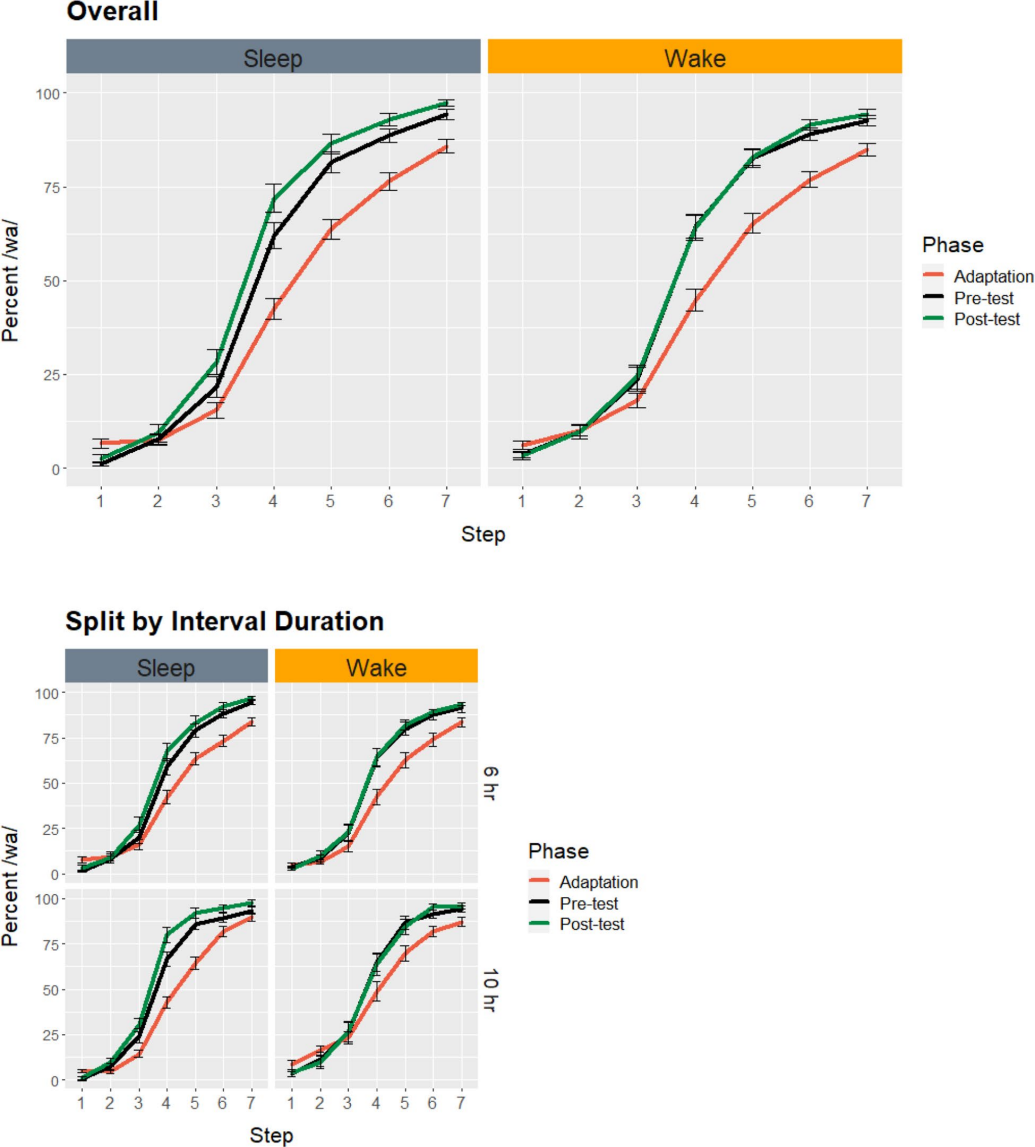
Average identification function at pretest, during adaptation, and at posttest, as a function of sleep, overall and split by interval duration. Error bars show standard error.

Figure 3 presents the average identification functions for the pretest, adaptation test, and posttest phases, separately for the Sleep and Wake groups. As can be seen, participants systematically identified the members of the /ba/ – /wa/ continuum, with the percentage of “wa” reports increasing from the endpoint /ba/ token (Step 1) to the endpoint /wa/ token (Step 7). There was robust selective adaptation, with “wa” reports being substantially reduced during the adaptation test (red curves) compared to the pretest (black curves).

To quantify the adaptation effect, and its change over the following 6 h or 10 h, we computed for each participant the percent “wa” report for the three middle items of the test continuum (i.e., Steps 3–5), getting this value for the pretest, adaptation test, and posttest data (see Footnote 1). We first analyzed the Pretest data to see whether the Sleep and Wake groups were matched in terms of how they heard the test continuum. This was clearly the case: A two-way ANOVA on the percentage of “wa” report at Steps 3–5 by participant showed no effect of group (55.1% [17.8] for Sleep vs. 56.9% [18.9] for Wake; *F* < 1), or interval duration (54.3% [18.8] for 6-h vs. 59.1% [17.4], for 10-h); *F*(1,103) = 1.74, *p* = .190, η^2^_G_ = .017, or their interaction (*F* < 1; see Table 1). An analysis of Bayes Factors using default priors in JASP (Version 0.19.3; JASP Team, 2024) found evidence for the absence of a difference between the two groups (BF_01_ = 4.37, *moderate*), or of an interaction (BF_01_ = 32.12, *very strong*; see Lee & Wagenmakers, 2013).

**Table 1:** Percentage of “wa” reports at pretest (standard deviations in brackets).

	Sleep group	Wake group
6 h	52.8 (18.8)	55.6 (19.0)
10 h	59.1 (15.7)	59.2 (19.2)

**Fig. 4:**
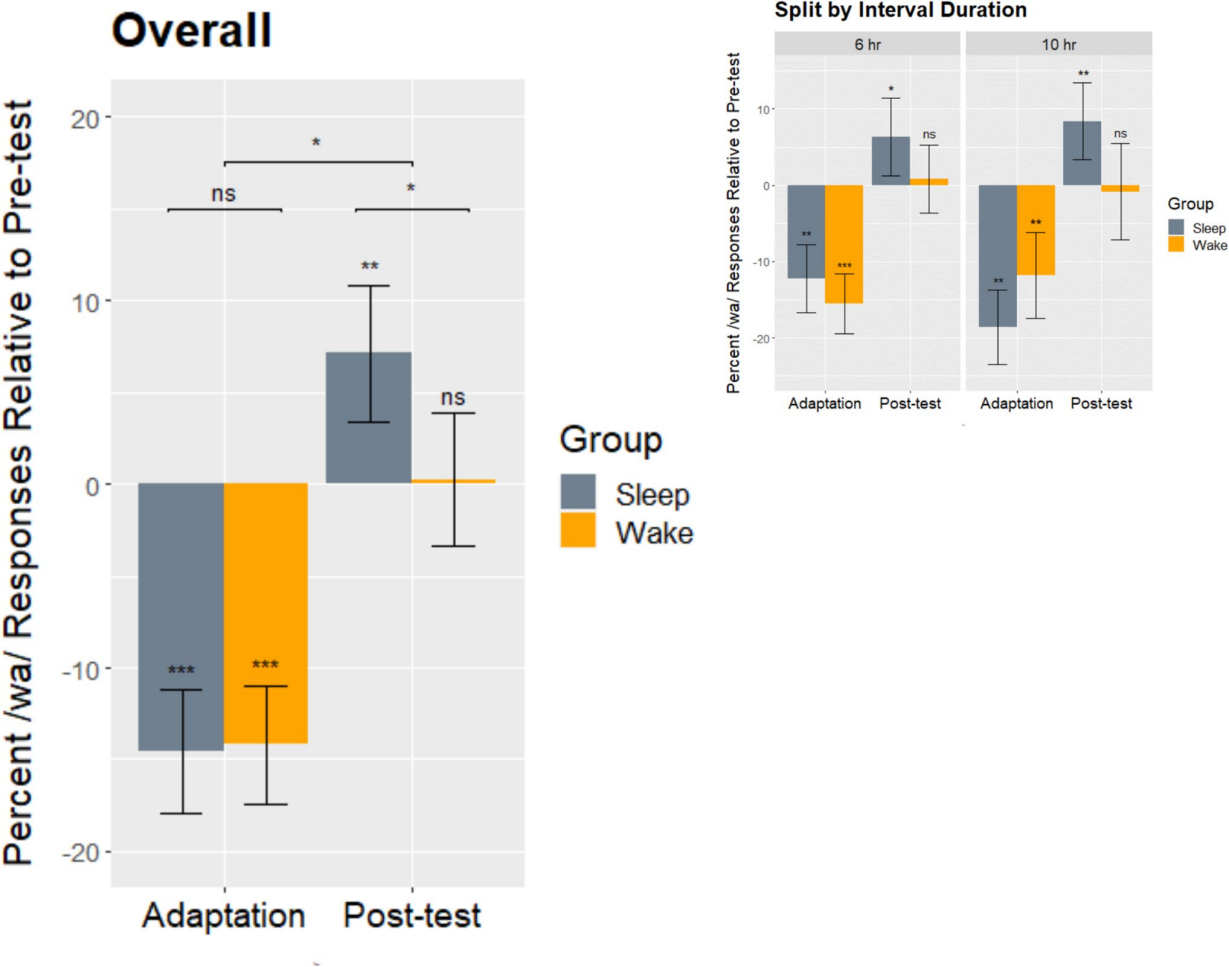
Change from baseline for the adaptation test (left side of each panel) and the posttest (right side of each panel), as a function of sleep, overall and split by interval duration. The plotted values are based on the percent “wa” report averaged across Steps 3–5. Error bars show standard error. The ***, **, and * symbols above the error bars refer to simple effects significant at, respectively, *p* < .001, .01, and .05; in the main figure, the * symbols the above horizontal brackets refer to two- and three-way interactions significant at *p* < .05. “ns” denotes nonsignificant.

Our main analysis tested both the immediate effect of adaptation (i.e., adaptation against pretest) and its long-term byproduct (i.e., posttest against pretest), as a function of the presence of sleep in the post adaptation interval. Figure 4 shows the corresponding difference scores in the percentage of “wa” report at Steps 3–5 collapsed across the 6-h and 10-h variations (main panel) or split by interval duration (inset). In each panel, the left side plots the difference from pretest baseline to adaptation; the right side plots the difference from baseline to posttest. (See Fig. S1 in the Supplementary Materials for difference scores at each step.)

As shown by an ANOVA for repeated measures with group (Sleep/Wake) and interval duration (6 h/10 h) as between-subject factors, the immediate effect of adaptation was highly reliable, *F*(1,103) = 46.42, *p* = .0001, η^2^_G_ = .149, and was not statistically different between the Sleep and Wake group (− 14.6% vs. − 14.2%, respectively; *F* < 1; Fig. 4, left-side bars). This effect was also not modulated by the interval duration (*F* < 1) or by a three-way interaction, *F*(1,103) = 1.40, *p* = .239, η^2^_G_ = .005. The equivalence between the two groups in terms of initial adaptation was confirmed by Bayesian evidence (BF_01_ = 15.46, *strong*), and so was the absence of interaction with interval duration (BF_01_ = 31.79, *very strong*) or of a three-way interaction (BF_01_ = 997.02, *extreme*). In sum, our Sleep and Wake groups were matched for initial identification of the test items, and also for the immediate influence of the adaptor, thereby showing no hint of time-of-day fluctuations. This matching provided ideal conditions to look at the effect of sleep on selective adaptation.

The right-side bars of Fig. 4 show that the Sleep group had a different postadaptation trajectory compared to the Wake group. This was attested by a significant interaction between group (Sleep/Wake) and test (Pretest/Posttest), *F*(1,103) = 6.09, *p* = .015, η^2^_G_ = .009. As supported by Bayesian evidence, by the posttest, adaptation was gone for the Wake group—identification curves had completely shifted back to Pretest (baseline) levels, with an average difference of only + 0.2%, *F*(1,56) = .0000008, *p* = .999, η^2^_G_ = .0004 (BF_01_ = 5.06, *moderate*).^2^ In contrast, adaptation in the Sleep group translated into a surprising and reliable shift into *positive* values compared with pretest levels, with 7.1% *more* “wa” report at posttest compared with baseline, *F*(1,47) = 10.25, *p* = .002, η^2^_G_ = .038. As the absence of a three-way interaction with interval duration indicates (*F* < 1, BF_01_ = 47.47, *very strong*), the effect of sleep on the postadaptation trajectory was the same for both the 6-h and 10-h variations of the protocol. The positive shift was significant for *both* the 6-h Sleep group, *F*(1,30) = 4.20, *p* = .049, η^2^_G_ = .026, and the 10-h Sleep group, *F*(1,17) = 8.97, *p* = .008, η^2^_G_ = .076. Confirming that the pretest to posttest change was tied to the presence of sleep in the interval rather than to mere differences in adaptation between the two groups, the interaction between group (Sleep/Wake) and effect (i.e., adaptation minus pretest vs. posttest minus pretest) was significant, *F*(1,103) = 5.41, *p* = .022, η^2^_G_ = .015.

## Discussion

Our results show that sleep neither stabilizes nor strengthens the perceptual shift that results from massed exposure to the same acoustic stimulus. Instead, after sleep, the adaptor’s category expands (see the green vs. the black curves in Fig. 3), reflecting its new frequency in the listener’s environment. This opposite-direction shift was quite unexpected: Adaptation effects have been characterized as a momentary adjustment due to fatigue (Eimas & Corbit, 1973), a shift in decision criterion (Diehl et al., 1978, 1980), or as a tightening of the long-term distribution of a phonemic category (Kleinschmidt & Jaeger, 2015, 2016). As Fig. 4 shows, repeated exposure to the adaptor has short-term consequences (captured by all three models) that are quite different from its longer-term consequences in the presence of sleep: Whereas in the short-term listeners do not perceive speech as belonging to the adaptor’s category as much as before adaptation, after sleep they perceive it as such even more than before adaptation (see Gaskell et al., 2014, for a nap study showing similar sleep-associated changes to the phonotactic representations used in speech production).

This pattern raises the question of whether immediate reduction of the adaptor’s category and its subsequent overnight expansion reflect two time points in the trajectory of the same learning phenomenon, or instead two independent processes running in parallel, both triggered by adaptor exposure. In the first of these scenarios, adaptation would be the precursor to category expansion: the stronger the adaptation, the stronger the postsleep rebound. In the second scenario, in contrast, with the two effects going in opposite directions, strong initial adaptation would simply mask the reverse identification shift (relative to pretest) that sleep produces. The first scenario predicts a negative correlation between the adaptation and the expansion effect for the Sleep group (i.e., a strong [negative] adaptation shift yielding a strong [positive] rebound); the second scenario predicts positive correlations. The correlations are in fact positive and of similar strength for the Sleep, *r*(47) = .48, *p* = .0005, and Wake, *r*(56) = .42, *p* = .001, groups (see Fig. S2 in the Supplementary materials). This argues against the notion that the overnight expansion effect merely carries over from selective adaptation, as posited in the single-process account.

These results, implicating two processes, cannot be accounted for by either classic adaptation theory (Diehl et al., 1978, 1980; Eimas & Corbit, 1973). Importantly, they also are inconsistent with Kleinschmidt and Jaeger’s (2015) prominent Bayesian account of speech plasticity. As we saw in the Introduction, these authors explain both attractive (i.e., recalibration) and contrastive (i.e., adaptation) perceptual after-effects through a single statistical learning mechanism shaping and reshaping phonemic categories to continuously reflect the distributional properties of the input (e.g., Clayards et al., 2008; Feldman et al., 2009; Maye et al., 2002). As such, this account therefore makes no provision for the observed, *postsleep* reverse adaptation shifts.

As we noted above, each of three prior models of selective adaptation relies on a single mechanism, compromising its ability to account for effects going in opposite directions. Our results strongly suggest that a model must have two separate mechanisms, one to produce the basic (contrastive) adaptation shift, and one to produce the (assimilative) increase found after sleep. Snyder et al. (2015) describe a Bayesian model that may offer the necessary two-factor properties. They review a large body of research on what they call “temporal context effects,” in multiple sensory modalities, in which perception of a stimulus is affected by previously experienced stimuli. They draw upon examples in vision (e.g., perception of Necker cubes; tilt illusions) and audition (e.g., auditory stream segregation; recalibration). Relying in part on neuroimaging work by Schwiedrzik et al. (2014), they posit that one process generates the observed contrastive shifts, while a completely separate process is responsible for the assimilative ones. The contrastive effect (here, the classic “adaptor category reduction”) is grounded in sensory changes that are similar to those in the original Eimas and Corbit (1973) sensory fatigue/habituation model. This shift typically occurs when an ambiguous target stimulus is preceded by a nonambiguous salient stimulus (e.g., as in our adaptation procedure). Neuroimaging evidence supports these effects as being generated in early perceptual regions (Schwiedrzik et al., 2014). The assimilative effect is described instead as a change in a category’s “priors”, exactly as would be expected by hearing hundreds of repetitions of an adaptor. These changes are associated with higher-level brain regions, ones with a longer time scale than those in sensory areas. Snyder et al. suggest that the contrastive effect allows the perceptual system to be sensitive to new information in the environment, while the assimilative effect enhances perceptual stability. Any model that has two mechanisms that produce opposing effects could, in theory, predict any result, and thus be unfalsifiable. In this case, the two mechanisms differ in time course, brain localization, and triggering stimulus attributes; these three distinguishing properties are consistent across vision and audition. Thus, the model is actually relatively well constrained in its predictions and is thus falsifiable.

The work by Snyder et al. (2015) and Schwiedrzik et al. (2014) is not specifically focused on selective adaptation in the speech domain, but it seems to provide a promising framework to account for our results. To a first approximation, their contrastive process is akin to Eimas and Corbit’s (1973) original account of adaptation, and their assimilative process is similar to the mechanism in Kleinschmidt and Jaeger’s (2015) account of recalibration shifts. The association of sleep-based consolidation with the higher-level assimilative process, and not with the lower-level contrastive one, is speculative, but plausible. The results of the current study provide an important impetus for theoretical and empirical development of these ideas.

## Supplementary Information

Below is the link to the electronic supplementary material.

Supplementary file1 (DOCX 114 kb)

## Data Availability

Our stimuli and data are publicly available through the OSF platform: https://osf.io/wakgy/

## Footnotes

1 In the current study, our statistical analysis follows the procedures we and others have used in dozens of papers (e.g., Samuel, 1988, 2016; Samuel & Dumay, 2021). The advantages of these procedures are described in Samuel and Dumay (2021). Briefly, this approach takes into account that adaptation effects are typically most robust near the middle of the test continuum, by design; this is also the region in which the observed values are far from the values that become nonlinear for proportions. By averaging the values for several items in the most sensitive region, the analysis maximizes power to detect any effect (or differences in effects across conditions). It also avoids mistakenly treating the successive steps of the test continuum as separate items (as would be done, for example, with mixed effects modeling). Studies using a test continuum really only have a single item (the test continuum), with the members of the continuum, by design, being nonindependent.

2 Samuel and Dumay (2021) used monaural adaptors and test items, and found stronger adaptation within ear than between ear. After 5 h and 30 min, the shift was still significant for the within-ear case, but not for the between case. In the current study, the shift sizes are more similar to those for the between case, and are also not significant after 5 h and 30 min.

## Code availability

Our analysis and visualization r-scripts are publicly available through the OSF platform: https://osf.io/wakgy/

## Declarations

### Open practices statement

Our stimuli and data as well as our analysis and visualization r-scripts are freely accessible from the OSF platform: https://osf.io/wakgy/.
