# Supplementary material for: How sleep redraws phonemic categories after auditory selective adaptation
Source: Psychon Bull Rev. 2026 Jan 14;33(1):44. doi: 10.3758/s13423-025-02819-x (PMC12804317; doi:10.3758/s13423-025-02819-x)
Supplement: Supplementary file 1 — Supplementary file1 (DOCX 114 kb) [file 13423_2025_2819_MOESM1_ESM.docx]

**Supplementary materials⎯Figures S1 and S2**


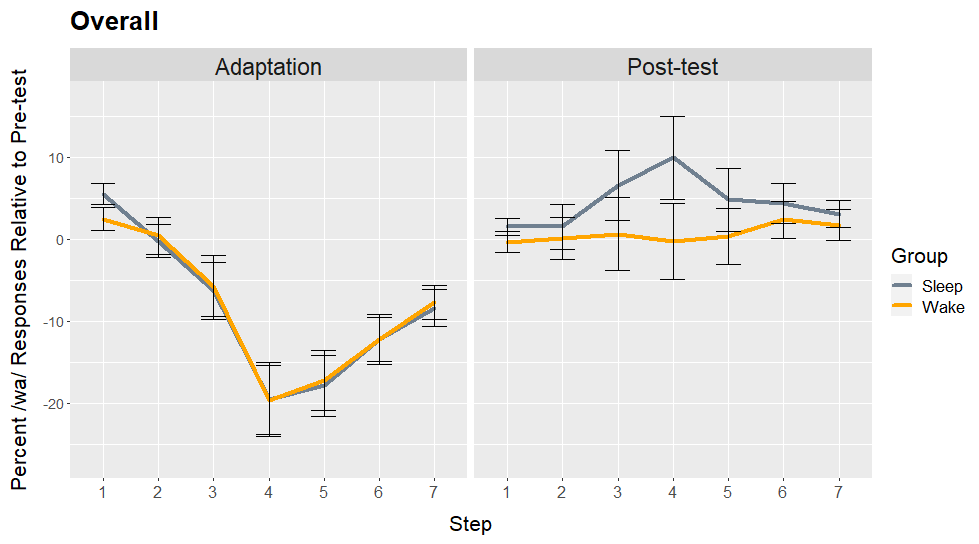


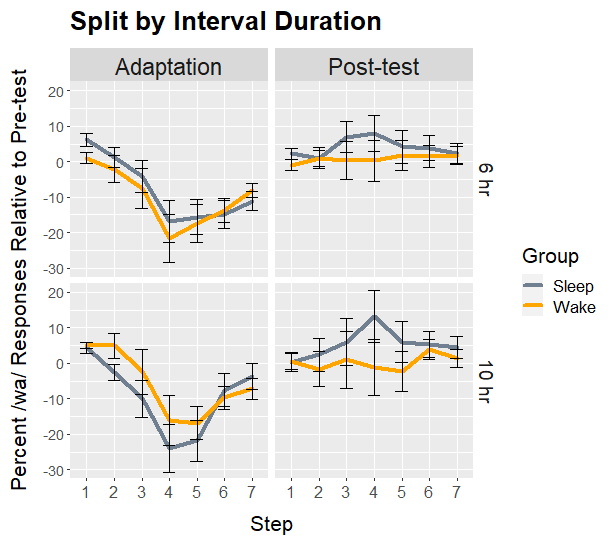


**Figure S1.** Change from baseline at each step of the continuum for the Adaptation test (left side of each panel) and the Post-test (right side of each panel), as a function of sleep, overall and split by interval duration. Error bars show standard error.


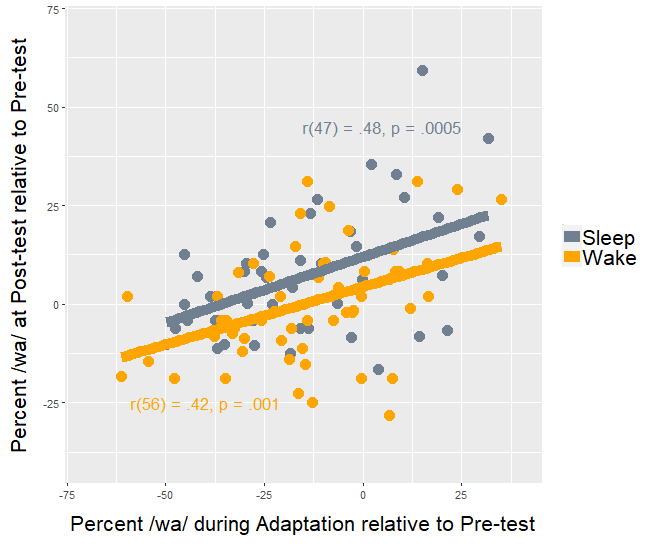


**Figure S2.** Pearson's correlation between adaptation and post-test in their difference scores of /wa/ responses relative to pre-test, separately for the Sleep and Wake groups, plus coefficients and regression lines.
